# Extracorporeal membrane oxygenation in paediatric cardiac surgery: 5-year single centre experience

**DOI:** 10.1186/s13019-023-02440-w

**Published:** 2023-11-10

**Authors:** Laurynas Bezuska, Jonathan P. O’Doherty, Bilal Ali, Chris Harvey, Ikenna Omeje, Branko Mimic

**Affiliations:** 1 East Midlands Congenital Heart Centre, University Hospitals of Leicester NHS Trust, Leicester, UK; 2 Clinic of Cardiac and Vascular Diseases, Institute of Clinical Medicine, Faculty of Medicine, Vilnius University, Santariskiu 2, Vilnius, LT-08661 Lithuania

**Keywords:** Extracorporeal membrane oxygenation, Paediatric, Cardiac ECMO

## Abstract

**Background:**

Extracorporeal membrane oxygenation (ECMO) has become an integral part of paediatric cardiac surgery. We report the experience of a well-established ECMO service over 5 years.

**Methods:**

This retrospective study analysed all paediatric patients who required ECMO support following cardiac surgery from April 2015 to March 2020. Inclusion criteria were age less than 18 years and post-operative ECMO support. Patients were analysed dividing into groups according to the urgency for ECMO support (extracorporeal cardiopulmonary resuscitation (ECPR) and cardiac ECMO) and according to age (neonatal and paediatric ECMO groups). They were followed for 30-day, 6-month mortality, long-term survival, postoperative morbidity and the need for reintervention.

**Results:**

Forty-six patients were included who had a total of venoarterial (VA) 8 ECMO runs. The 5-year incidence of the need for VA ECMO after cardiac surgery was 3.3% (48 of the overall 1441 cases recorded). The median follow-up period was 3.5 (interquartile ranges, 0.8–4.7) years. Thirty-day, 6-month and follow-up survival rate was 85%, 65% and 52% respectively. At the 6-month follow-up, the ECPR group showed a trend towards worse survival compared with the cardiac ECMO group (47% vs. 55%) but with no statistical significance (p = 0.35). Furthermore, the survival rates between paediatric (60%) and neonatal (46%) ECMO groups were similar, with no statistical significance (p = 0.45). The rate of acute neurological events was 27% (13/48).

**Conclusion:**

ECPR and neonatal ECMO groups had higher mortality. VA ECMO 30-day and 6-month survival rates were 85% and 65% respectively. Major neurological injury resulting in ECMO termination occurred in 3 patients. Accumulated experiences and protocols in ECMO management can improve mortality and morbidity.

## Introduction

Extracorporeal membrane oxygenation (ECMO) has been used to treat critically ill patients with respiratory and/or cardiac failure for 50 years. Hill and colleagues reported the first successful use of long-term extracorporeal life support in 1972 for a patient with acute post-traumatic respiratory failure [1]. In 1974 the first neonate, Baby Esperanza, successfully survived ECMO which was reported by Bartlett and colleagues [2, 3]. Published in 2009 the landmark CESAR trial definitively demonstrated the utility of ECMO. This multicentre randomised controlled study clearly showed an advantage of ECMO for patients with severe respiratory failure [4].

Nowadays, no good quality congenital cardiac unit can function without the backup of perioperative ECMO, which is often life-saving [5–8]. The main benefit of ECMO is to provide cardiac output and oxygenation while “resting” the heart and lungs following the operative insult. This allows time for recovery of function; this is ECMO as a “bridge to recovery”. Other indications include a bridge to a ventricular assist device and/or heart transplantation, extracorporeal cardiopulmonary resuscitation (ECPR), failure to wean from cardiopulmonary bypass or to stabilize preoperative patients before cardiac surgery [5, 8, 9]. Despite this broad spectrum of utility, up until now, there have been no widely agreed protocols for instituting ECMO due to high cost and complication rates.

The Extracorporeal Life Support Registry report distinguishes two separate groups for ECMO patients under 18 years old: neonatal and paediatric. Survival outcomes are reported by dividing patients into pulmonary, cardiac or ECPR groups [10].

Recent studies suggest the incidence of ECMO for children undergoing congenital cardiac surgery is 2 to 4% [6–8]. Khorsandi and colleagues described their 5-year paediatric experience with an ECMO rate of 4% and a survival rate to hospital discharge of 44% [6]. Mascio’s paper reports data from 80 USA centres with an average incidence of 2.4% for ECMO and in-hospital mortality of 53% [7]. Brown and colleagues reported data from 5 UK paediatric cardiac centres with an ECMO incidence rate of 2% and with 30-day and 6-month survival rates of 68% and 54% respectively [8]. In this article, we report 5 years of data from a well-established ECMO service.

## Methods

This is a retrospective study that analysed all paediatric patients at our institution who required ECMO support following cardiac surgery from April 2015 to March 2020. All patients were operated on at a single institution. Inclusion criteria were: age less than 18 years and the requirement for post-operative ECMO support. All included patients had venoarterial (VA) ECMO support. Patients were identified from the ECMO database which contains operative, perioperative, and outcome data. Further clinical details from admission until the last follow-up were obtained from the “HeartSuite” database, electronic and paper medical notes. It was checked by two assessors (LB and JO). Seven cases were excluded: four patients underwent catheter-related procedures, one child had respiratory ECMO before coarctation of aorta repair and two patients had preoperative stabilization on ECMO without the need for postoperative ECMO.

The patients were divided into two groups according to the urgency for support: ECPR or cardiac ECMO. Additionally, for analysis, patients were further separated into two separate groups according to age: neonatal (up to 30 days of age) and paediatric (from 31 days to under 18 years of age).

In all patients, the consent for surgery and the utilization of data for publication and/or presentation in scientific meetings was signed by one of the parents or by the legal guardian the day before surgery. This project did not need to be submitted to a Research Ethics Committee for ethical approval as it was a clinical audit authorized by the University Hospitals of Leicester NHS Trust (audit number: 10,072).

### ECMO protocol

The ECMO post paediatric cardiac surgery is initiated in the event of failure to wean from cardiopulmonary bypass, low cardiac output or severe hypoxemia not responding to conventional treatment in the first days after surgery and ECPR. Central chest cannulation is our favoured approach for post-cardiotomy ECMO. In cases where ECMO becomes necessary due to unsuccessful weaning from bypass, connecting the circuit to the existing cannulas is typically a more straightforward procedure. Adequate attention must be given to ensuring the cannulas remain firmly in place and that the applied purse strings effectively control bleeding. If extended ECMO support is anticipated, peripheral cannulation is recommended. This offers more reliable support, reduces the risk of bleeding, and allows for the chest to be formally closed.

Protamine is routinely administered upon coming off cardiopulmonary bypass to manage bleeding. In our institution, we implement ECMO running protocols with low or no heparin usage, particularly in cases of significant bleeding. The optimization of ECMO circuit design aims to minimize thrombogenicity (“Short circuit, minimal connections”). In the event of bleeding, aggressive product management is guided by the thromboelastogram. Our standard anticoagulation policy includes intravenous heparin infusion with a target activated clotting time in the range of 180–200 s and the test being repeated every 4 h.

### Definitions

Thirty-day and 6-month mortality were defined as death occurring within 30 days and 6 months post-ECMO decannulation respectively. Follow-up mortality was counted as 30-day and 6-month mortality combined with follow-up. Neurological complications were defined by Brown and colleagues [8] as acute neurological events that include any new neurological morbidity around the time of ECMO and are diagnosed either by electroencephalogram, brain scan (computed tomography or magnetic resonance) or by clinical evaluation.

### Data analysis

The statistical software SPSS 26.0 for Windows (SPSS Inc. Chicago, Illinois, USA) was utilised. Data are presented as medians and interquartile ranges (IQR) due to the non-normal distribution of variables. The Kaplan-Meier method was used to evaluate survival functions. A value of p < 0.05 was considered to be significant.

## Results

**Fig. 1:**
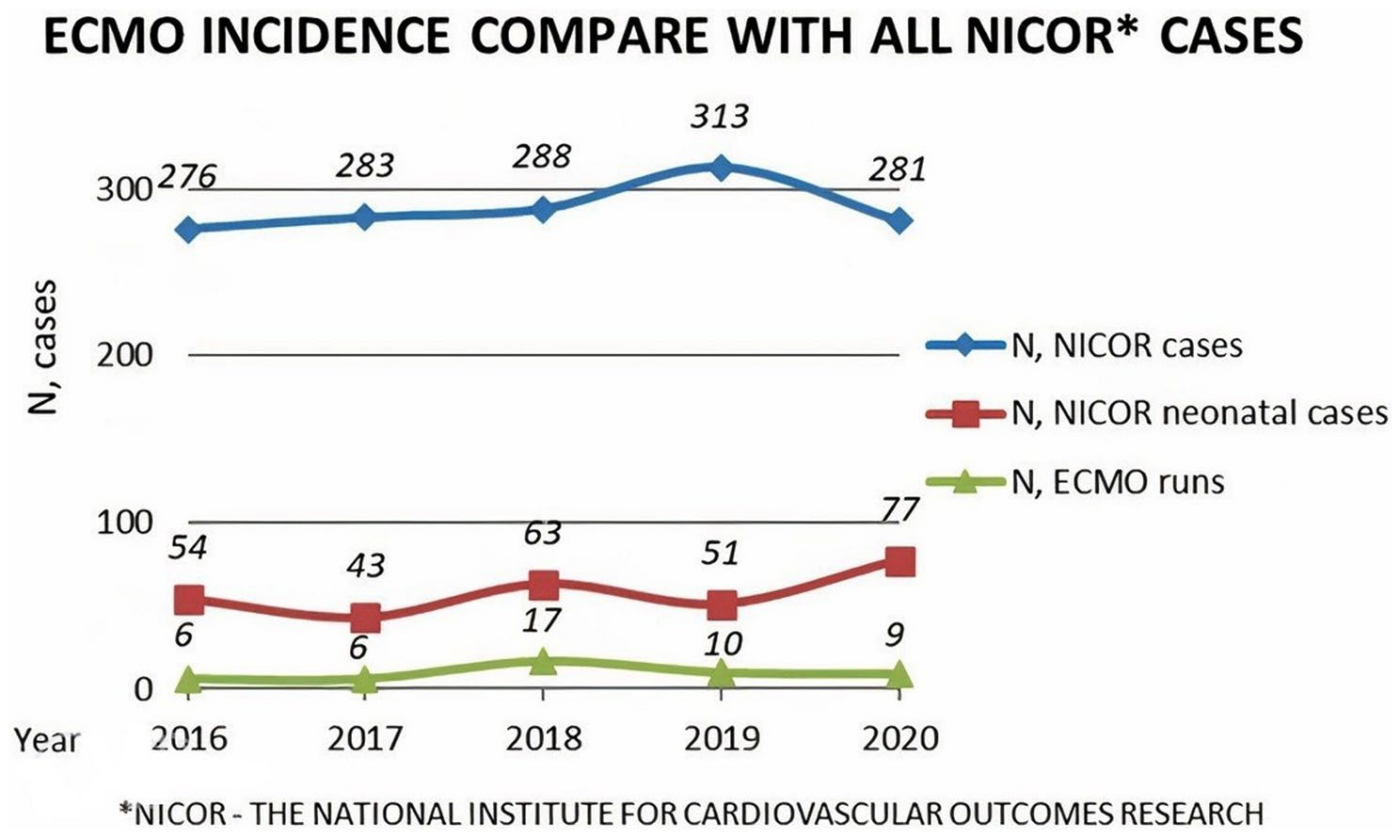
ECMO incidence compared with neonatal and all NICOR cases per 5-year period. NICOR – The National Institute for Cardiovascular Outcomes Research

A total of 48 venoarterial (VA) ECMO runs or 46 patients younger than 18 years were included, 28 (61%) of them were neonates. Two patients had two ECMO runs; in both cases, the second run was necessitated by severe hypoxia unresponsive to conventional treatment. The median VA ECMO run duration was 5 (IQR, 3–11) days and the median follow-up time was 3.5 (IQR, 0.8–4.7) years. Over the 5 years, the ECMO rate was 3.3% (48/1441) averaging just under 10 ECMO runs per year. ECMO incidence compared with neonatal and all NICOR cases per 5-year period is shown in Fig. 1. Median weight was 3.7 (range: 2 to 41.8) kg.

The most common procedure needing VA ECMO support was a Norwood-type operation (27%). An arterial switch operation was the second most common surgery with 6 children (13%) requiring ECMO; all these patients except one required concomitant procedures such as ventricular septal closure or aortic arch repair. ECMO support was needed for 5 patients (10%) after aortic arch repair with a concomitant cardiac procedure, these were: 2 ventricular septal defect closures, Glenn procedure, cor triatum repair and atrial septectomy with pulmonary artery banding. The full list of procedures is shown in Table 1.

**Table 1:** ECMO incidence by operation type

Procedure	N, patient	Fraction of ECMO runs
Norwood type procedure	13	27%
Arterial switch operation	6	13%
Aortic Arch repair with a concomitant cardiac procedure	5	10%
Shunt procedure	4	8%
Isolated pulmonary artery banding	3	6%
Fontan procedure	2	4%
Glenn procedure	2	4%
Atrioventricular septal defect repair	2	4%
Arterial trunk repair	2	4%
Anomalous left coronary artery from the pulmonary artery repair	1	2%
Total anomalous pulmonary venous connection repair	1	2%
Tetralogy of Fallot repair	1	2%
Yasui procedure	1	2%
Double outlet right ventricle repair	1	2%
Ross + Konno procedure	1	2%
Mitral valve replacement	1	2%
Ventricular septal defect patch disintegration repair	1	2%
Supravalvular aortic stenosis repair and pulmonary artery plasty	1	2%

Thirty-day, 6-month and follow-up survival rates were 85%, 65% and 52% respectively. Table 2 illustrates the ECMO survival rate per 5-year period with a comparison to other centres analysing paediatric cardiac ECMO data.

**Table 2:** ECMO survival by years comparing data with other centres

Study Year	N, patient	N, ECMO run	30-day Survival, %	6-month survival, %	Follow-up survival, %
2016	6	6	100	50	50
2017	6	6	83.3	83.3	83.3
2018	16	17	88.2	70.6	52.9
2019	9	10	80.0	50.0	40.0
2020	9	9	77.8	66.7	44.4
**TOTAL**	**46**	**48**	**85.4**	**64.6**	**52.1**
*Scotland*^*6*^: 2011–2016	*66*	*66*	*45* ^*^	*44*	*44*
*USA*^*7*^: 2000–2010	*NA*	*2318*	*53.2* ^*^	*NA*	*NA*
*UK*^*8*^: 2015–2017	*57*	*62*	*68.4* ^*^	*54.4*	*NA*

*NA – not available*

* – The study states the discharge or transfer survival rather than the 30-day survival

**Fig. 2:**
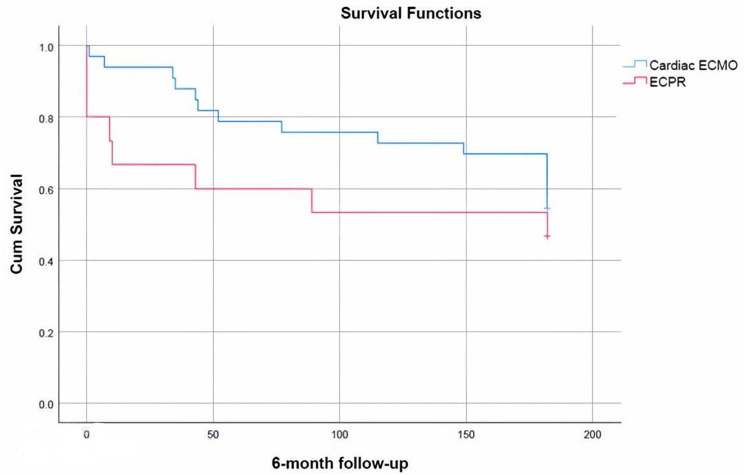
ECPR compared with cardiac ECMO group survival functions

**Fig. 3:**
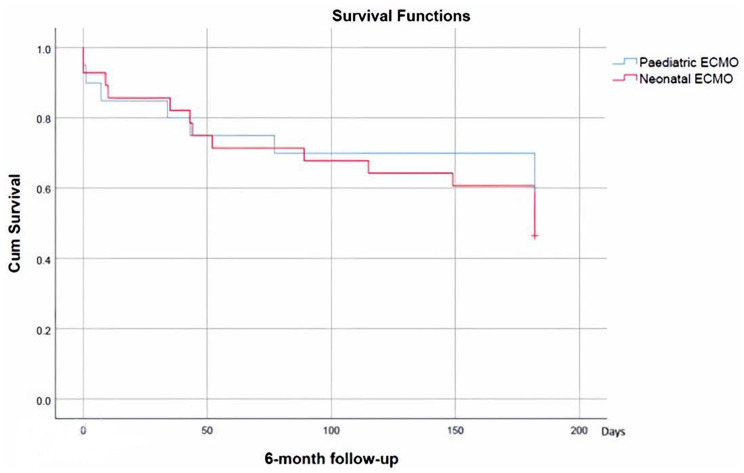
Paediatric and neonatal ECMO groups survival functions

ECPR compared with the cardiac ECMO group showed no statistically significant difference in survival (47% versus 55% and log-rank p = 0.35), but with a trend towards worse survival in the ECPR group (Fig. 2). Categorised by age 28 neonatal and 20 paediatric ECMO runs were analysed. Paediatric and neonatal ECMO groups had similar survival rates (60% versus 46%) without a statistically significant difference (log-rank p = 0.45). The survival function for this group is shown in Fig. 3.

Twenty-six ECMO runs were managed as a bridge to recovery with seven patients of this group having diagnostic catheter procedures without the need for the intervention. While the rest 22 ECMO runs were bridged to interventions with 20 surgical and two interventional catheter procedures. The initial decision for ECMO was destined for recovery, but some above-mentioned cases required intervention.

### Complications

In total, we have observed 13 acute neurological events (13/48, 27%) which is in line with high-capacity centres (11). Neurological morbidities were confirmed by computed tomography of the brain in eight patients, magnetic resonance imaging of the brain in two children and clinical evaluation in two children. Major neurological injury resulting in ECMO withdrawal occurred in 3 patients (3/48, 6%).

Acute kidney injury requiring renal replacement developed in 22 (46%) patients; none of them requiring long-term dialysis. Nine (19%) children required re-exploration for major bleeding. Three patients (6%) suffered from necrotic enterocolitis with one infant requiring laparotomy. Two patients had their arterial ECMO cannulas accidentally dislodged resulting in a period of cardiac arrest and replacement of the ECMO cannulas.

### Mortality

Seven patients (7/48, 15%) did not survive 30 days. Four of them were neonates. ECMO was discontinued for two babies due to severe intracranial haemorrhages and for one child due to severe hypoxic-ischaemic injury of the brain. One neonate failed to wean from ECMO due to multiorgan dysfunction syndrome. Whilst waiting for the Norwood procedure, she had an acute deterioration with sudden profound metabolic acidosis necessitating ECPR and palliative pulmonary artery banding. ECMO support was withdrawn for one more child due to severely impaired ventricular function as heart transplant units excluded the child due to comorbidities and long (over 2 weeks) ECMO run. Another neonate died nine days after ECMO weaning due to multiorgan dysfunction syndrome. Importantly, additional information from computed tomography imaging demonstrated abnormal coronaries with fistulae connecting the left coronary artery and right ventricle. Another child died 7 days after weaning from ECMO post-Glenn procedure due to severe and acute desaturation which precluded any other interventions and rendered ECMO unsuitable.

Ten patients died between the 30-day and 6-month period. Three patients passed away due to severe chest infection. Two children experienced cardiac arrests in the community with unsuccessful resuscitation. One child died at the referred transplant centre due to severe left heart failure while waiting for a donor. Right ventricular perforation during the catheter procedure was a reason for another death. One more patient had severe tracheobronchomalacia and required long-term invasive ventilation, and suffered an untreatable arrhythmia. Persistent severe pulmonary hypertension was the cause of another death. The last child died due to cardiac arrest after prolonged treatment in the general intensive care unit.

There were 6 late deaths during the follow-up period. The initial diagnoses for these patients were as follows: tricuspid atresia with transposition of the great arteries, hypoplastic left heart syndrome, double outlet right ventricle (with severe non-cardiac comorbidities), hypoplastic aortic arch with ventricular septum (with severe non-cardiac comorbidities), and two patients had hypoplastic aortic arch with severe mitral stenosis. One patient passed away due to severe right heart failure complicated by cardiac arrest. One child died after a concomitant Glenn procedure which was complicated by severe cardiopulmonary failure. Another child’s cause of death was chronic severe chylothorax and cardiopulmonary failure. One patient died due to extensive intracranial haemorrhage after a catheter procedure for dilation of the stent in the innominate artery. The reason for one child’s death outside the hospital was not clear.

## Discussion

Rates of ECMO incidence in paediatric cardiac surgery and outcomes have remained similar across the past decade [6–8, 11]. The rate of ECMO incidence can vary quite significantly even not taking into consideration the surgical volume of a unit [7]. One can speculate that the rate has not increased with time due to improved diagnostic modalities and surgical techniques. A decision for cardiac ECMO is typically made on an empirical judgment and case-by-case basis; there are currently no internationally agreed protocols [6–8]. At our institution, every deteriorating patient after cardiac surgery is considered an ECMO candidate. Reversibility of cardiac pathology and a prompt diagnosis are key factors for success. However, the cost of ECMO and the high rate of complications should also always be borne in mind.

We reported a rate of neurological events higher than some other similar studies [6, 7]. However, these studies have reported only major neurological injuries [6, 12]. We wanted to include any neurological event related to ECMO as per the definition by Brown and colleagues [8]. According to this definition, our neurological complication rate is similar to high-capacity centres [8, 13]. Our rate of neurological events was 27%, but it included only three major neurological injuries (6%) resulting in ECMO termination. Lorusso and co-authors advocate the importance of brain monitoring with early and late assessments as it is becoming increasingly evident that neurological injuries may not occur only in the early phase [14].

According to the literature, bleeding requiring re-exploration occurs in up to a third of the patients [6, 7, 15, 16]. It has a negative impact on survival [6]. Our study reported a 19% incidence of severe bleeding. The lower rate of bleeding could be explained by our institution’s utilization of low or no heparin ECMO running protocols which are applied in the case of significant bleeding.

Another common ECMO complication is necrotic enterocolitis [6, 7, 17]. Khorsandi’s study reports an 18% incidence of this complication [6]. Capriati advocates the importance of preventing ischemia and necrosis at the level of the bowel wall and modifying the diet to prevent this complication [18]. Our reported patients had a relatively low incidence of necrotic enterocolitis (6%) and with only one patient requiring laparotomy. This could be partially explained due to the high-risk feeding protocol developed in our institution. A Sano shunt, as the preferred blood supply for the Norwood operation in our institution, is beneficial in reducing diastolic steal and the risk of necrotic enterocolitis. Another reason could be a prompt ECMO implementation in a deteriorating child preventing a long period of hypotension.

We reported our early survival rate as 30-day survival in line with the standard report of the National Institute for Cardiovascular Outcomes Research. Our reported 85% survival rate is higher compared to other centres [6–8]. We consider a 6-month survival rate to be a more definitive outcome as compared to survival to discharge or transfer. Once again, our reported 65% survival rate was better compared to other centres (Table 1) or data from the Extracorporeal Life Support Organization registry [10]. Barrett with colleagues associated paediatric cardiac surgical volume with mortality after cardiac ECMO [19]. The authors stated cardiac ECMO survival is better in centres that yearly performed ≥ 7 ECMO runs and ≥ 158 cardiac surgical cases.

In our study, we did not observe any significant improvement in the survival rate over the course of the five-year period. (Table 1). A higher number of ECMO runs per year could be partially explained by a higher number of neonatal surgeries per given year. All our patients were initiated on VA ECMO, as this is our preferred institutional approach for post-cardiotomy patients.

Our centre does not provide heart and/or lung transplantation services. ECMO was the only means of mechanical circulatory support (MCS) at our institution. While ECMO is still by far the most widely used form of MCS in paediatric cardiac surgery, it has recently been joined by alternative devices for univentricular or biventricular support [20, 21].

Being the first ECMO centre in the UK, our institution has accumulated many years of experience [14]. We provide both respiratory and cardiac ECMO services. For this reason, our ECMO team is quite confident in initiating ECMO and managing the circuit. This could partially explain the slightly higher rate of ECMO incidence in our institution (3.3%) compared with some other centres [7, 8]. The enhancements in cannula design and ECMO circuitry, along with the proactive use of ECMO before the onset of end-organ damage rather than in the final stages, have yielded favourable outcomes for postoperative survival and potential morbidity.

Currently, an effort is being made to develop a model for survival after paediatric cardiac VA ECMO. Geisser with colleagues developed the Pedi-SAVE Score after analysing 15-year data from the Extracorporeal Life Support Organization registry [22]. Non-single ventricle congenital heart disease, older age, white race, lower STAT mortality category, higher pH, not requiring acid-buffer administration, and < 2 cardiac procedures were pre-cannulation factors associated with survival. While lower ECMO pump flows at 24 h and lack of complications were post-cannulation factors associated with a better outcome. This score is a novel tool that can help in prognosis and counselling.

### Study limitations

A retrospective nature and a relatively small number of patients are limiting factors for this single institutional study. The more contemporary patients have limited follow-up so late morbidity and mortality may be underrepresented.

## Conclusion

ECPR and neonatal ECMO groups had a trend of higher mortality with no statistical significance. The 30-day and 6-month survival rates for VA ECMO stood at 85% and 65%, correspondingly. Three patients experienced significant neurological complications necessitating ECMO discontinuation. The accrual of expertise and adherence to ECMO protocols can lead to enhanced outcomes in terms of both survival and morbidity.

## Data Availability

The datasets used and analysed during the current study are available from the corresponding author upon reasonable request.
